# The effects of self-perceived aging and emotion regulation strategies on psychological abuse of elderly people in rural China: a structural equation modeling approach

**DOI:** 10.3389/fpsyg.2025.1569023

**Published:** 2025-06-13

**Authors:** Li Pei, Dongqing Zhao, Futing Cao, Shuang Li, Lanrui Zhang, Xiaomeng Wu, Xiaoli Pang, Haoying Dou

**Affiliations:** ^1^School of Nursing, Tianjin University of Traditional Chinese Medicine, Tianjin, China; ^2^Graduate School, Tianjin University of Traditional Chinese Medicine, Tianjin, China; ^3^School of Pharmacy, North China University of Science and Technology, Tangshan, Hebei, China

**Keywords:** elder abuse, aged care, cognitive reappraisal, psychological abuse, rural

## Abstract

**Background:**

In China, psychological abuse is increasingly prevalent among elderly people. Psychological abuse can have a significant negative impact on elderly people in terms of worsening chronic illness, increased suicide rick and death. Previous studies have suggested that self-perceived aging may serve as a risk factor for psychological abuse. However, the immediate impact of self-perceived aging on psychological abuse within Chinese culture remains unclear.

**Objectives:**

This study aimed to investigate the relationship between self-perceived aging and psychological abuse among rural Chinese older adults, while considering the parallel mediating role of emotion regulation strategies (including cognitive reappraisal and expressive inhibition).

**Research design and methods:**

A cross-sectional design was used in this study. A total of 449 rural older adults were recruited from a county in Dezhou City, Shandong Province, from July to October 2023 to assess self-perceived aging, cognitive reappraisal, expressive inhibition and psychological abuse. A hypothesized model based on Socioemotional Selectivity Theory pathways was proposed to examine the relationships between self-perceived aging, cognitive reappraisal, expressive inhibition and psychological abuse.

**Results:**

Higher self-perceived aging and expressive inhibition were positively associated with the propensity to be psychological abuse, and cognitive reappraisal was negatively associated with psychological abuse. Structural equation modeling revealed that the cognitive reappraisal and expressive inhibition strategy mediated the relationship between self-perceived aging and psychological abuse.

**Discussion and implications:**

This study reveals that self-perceived aging is associated with the occurrence of psychological abuse in older adults as they enter the aging stage. It further suggests that part of this effect can be explained by cognitive reappraisal or expressive inhibition. Thus, the use of emotion regulation strategies may help to reduce the incidence of psychological abuse after self-perceived aging.

## 1 Introduction

China hosts one of the largest aging populations globally, with rural communities—representing 33% of the population in 2024: facing unique challenges in elderly care (National Bureau of Statistics of China, 2025). For a long time, rural areas in China have shown the remarkable population characteristic of “getting old before getting rich” (Li et al., 2024). This special demographic and economic pattern not only subject the rural elderly population to severe pressure in terms of old-age security, but also gives rise to a series of social problems. Psychological abuse has been identified as a more prevalent yet frequently overlooked issue in rural areas (Gil et al., 2015). Identifying its associated risk factors represents a critical research priority.

Psychological abuse is the infliction of pain, psychological distress or suffering through verbal or non-verbal behavior (Hall et al., 2016). It is not limited to verbal aggression, but also includes isolating older people from family and friends, or preventing them from participating in everyday activities. The “silent treatment” can be extremely pernicious to psychological health (Conrad et al., 2010). The global prevalence of psychological abuse is relatively high. In a study of elder abuse in seven European countries, psychological abuse was identified as the most common form of elder abuse (Lindert et al., 2013). The results of a meta-analysis showed that the global rate of abuse was as high as 15.7%, with psychological abuse being the subtype with the highest incidence (Yon et al., 2017). The results of a decade-long longitudinal study in the United States indicated that the prevalence of psychological abuse of older adults within the family was 4.1% (Burnes et al., 2021). In China, research on elder abuse is still at an early stage, and there are relatively few studies on different types of elder abuse in different regions. Meta-analysis shows that at least 3 in 10 older adults in rural China have experienced abuse and neglect, and that psychological abuse is the type of abuse with the highest incidence in rural China (Zhang et al., 2022). Oluoha et al.'s comparative study on elder abuse in urban and rural revealed that 90% of rural elderly participants reported experiencing abuse: a significantly higher proportion than their urban counterparts. Remarkably, the incidence of psychological abuse among rural elders was three times greater than that in urban populations (Oluoha et al., 2017). These empirical data demonstrate that elder abuse is deeply entrenched in societal structures, with rural communities facing disproportionately higher risks due to systemic vulnerabilities. Additionally, in the context of traditional Chinese cultural values and the hidden and persistent nature of the occurrence of psychological abuse, its incidence may be underestimated. Notably, Chinese older adults are reluctant to disclose potential abuse and will choose to tolerate it in order to maintain family harmony (Zheng et al., 2019). Therefore, we presumably that the risk of elder psychological abuse may be more prevalent in rural China.

Currently, there remains a lack of comprehensive and in-depth understanding globally regarding the influencing factors associated with the psychological abuse of elderly individuals in rural areas of China. Significant research gaps exist in this field, and the relevant influencing factors have not been thoroughly explored. Fortunately, our team is committed to the study of psychological abuse of the elderly, and we have developed Elder Psychological Abuse Scale and studied the factors influencing the psychological abuse of elderly people in China (Wu et al., 2025). Other research groups have found that, based on the Socioemotional Selectivity Theory (SST), stronger personal coping skills may reduce the risk of prior psychological abuse. This risk may also prompt the elderly to actively build personal and family relationships, reducing the trauma caused by psychological abuse (Gruhn and Compas, 2020). SST suggests that perceived aging and inappropriate application of emotion regulation strategies may trigger the risk of abuse (Löckenhoff and Carstensen, 2004). Older persons often self-perceived aging as a threat, and ageism against older persons in the outside world further increases the risk of psychological abuse of older persons (Pillemer et al., 2016).

Self-perceived aging (SPA) refers to the subjective cognitive and psychological responses of older adults when they are threatened by physical, psychological, and social aging, and it affects behavioral tendencies in the aging process (Barker et al., 2007). According to the different views, aging can be perceived as either positive or negative (Gao et al., 2022). SPA can impact physical functioning, cognition, lifestyle behaviors, and healthy aging in older adults (Pan et al., 2019). Elderly adults in a positive SPA are more likely to mitigate the negative effects of psychological abuse (e.g., isolation and disrespect) through greater psychological resilience and social support (Czibere et al., 2019; Wurm and Benyamini, 2014). Positive SPA will allow older adults to better control and plan their lives and adopt more preventive health behaviors to slow disease progression and increase mental resilience. Conversely, Older adults in a negative SPA state tend to refuse social support interventions, which diminishes their life engagement and affects psychological resilience, leading to the higher risk of psychological abuse. Therefore, we propose Hypothesis 1.

Hypothesis 1: Self-perceived aging is related to psychological abuse.

The choice of emotion regulation (ER) strategies is a significant factor influencing psychological abuse among older adults. Individuals typically use expressive inhibition and cognitive reappraisal as emotion regulation strategies (Wang and Guo, 2003). Cognitive reappraisal occurs early in the emotion-producing process and deals with reinterpretation of the situation to minimize its psychological impact. Expressive inhibition occurs late in the process of emotion generation and involves inhibiting the outward expression of inner feelings (Gross, 2002). Nowadays, few studies have examined psychological abuse in relation to cognitive reappraisal and expressive inhibition among older adults in rural China. Moreover, existing research has mainly focused on adolescent or child samples. Adaptive use of emotion strategies can influence therapeutic outcomes for abuse-related psychological risks (Wooten et al., 2022). Andrei Ion's study revealed a correlation between decreased use of cognitive reappraisal and childhood abuse (Ion et al., 2023). Individuals with psychological abuse histories frequently exhibit emotion regulation difficulties, demonstrating a propensity for maladaptive strategies like expressive inhibiting. Intervention studies suggest that targeted emotion regulation training could potentially mitigate abuse incidence (Berzenski, 2019; Jennissen et al., 2016). Findings from Zhou and Zhen indicate that cognitive reappraisal may help adolescents attenuate the adverse emotional impacts of psychological abuse (Zhou and Zhen, 2022). Weissman et al. (2019) observed positive associations between abuse experiences and expressive inhibiting use.

Based on the Socioemotional Selectivity Theory (SST), when individuals feel that they are aging, their emotional regulation ability is likely to undergo significant changes. Since the elderly are aware of the shortening of the remaining lives, emotional goals ascend to the forefront of their priorities. This shift is manifested by an increased focus on their own emotional wellbeing (Wirth et al., 2023). This perception of aging will prompt them to re-evaluate negative life events from diverse perspectives. Fu et al. (2022) discovered that the elderly with a perception of negative SPA have difficulty in effectively dealing with and regulating their emotions. Negative SPA in older adults reinforce the use of expressive inhibition strategies. This maladaptive emotion regulation may subsequently increase vulnerability to psychological abuse. Although these links have been partially established, it remains to be verified whether emotion regulation strategies play a key mediating effect in self-perceived aging and psychological abuse in older adults.

Previous studies have demonstrated that two emotion regulation strategies, cognitive reappraisal and expressive inhibition, tend to play a mediating role, and most of the studies have been conducted with adolescents as subjects (Yin et al., 2022; Zhou and Zhen, 2022). Few empirical studies have integrated the four components of psychological abuse, self-perceived aging, cognitive reappraisal, and expressive inhibition to explore them as a comprehensive model of rural Chinese older adults. Therefore, the present study aimed to investigate the relationship between self-perceived aging and psychological abuse among rural Chinese older adults, taking into account the mediating roles of cognitive reappraisal and expressive inhibition. Based on the SST and available evidence, Hypotheses 2 and 3 are made.

Hypothesis 2: Cognitive reappraisal mediates the relationship between self-perceived aging and psychological abuse.
Hypothesis 3: Expressive inhibition mediates the relationship between self-perceived aging and psychological abuse.

## 2 Methods

### 2.1 Participants and procedure

The survey was conducted using a cross-sectional design in the rural area of Dezhou City, Shandong Province, China. It follows the guidelines for reporting of observational studies, the Strengthening the Reporting of Observational Studies in Epidemiology (STROBE).

From July to October 2023, we recruited 449 elderly participants through convenience sampling in rural areas of Dezhou, Shandong Province. Researchers conducted door-to-door visits in collaboration with local village committees to recruit eligible elderly participants. Researchers were trained to explain the purpose of the study to the older adults prior to the survey, and after seeking consent, a one-on-one, face-to-face questionnaire was administered to the older adults who met the criteria for nativity. In the case of illiterate older adults, the entries were read loudly by the researcher, the older adults responded, and the researcher recorded them. Use uniform guidelines and try to keep it to about 15 min. Elderly people fulfill the following requirements: (1) Reside within the communities of the county and are ≥60 years of age; (2) Communicate fluently and ability to fill out questionnaires with the help of the researcher; (3) Provide informed consent and voluntary participation. Exclusion Criteria: (1) Have lived in the county community for < 6 months; (2) Severe cognitive dysfunction, physical and psychological illness.

This study was approved by the Review Board (IRB) of Tianjin University of Traditional Chinese Medicine. Informed consent, voluntary participation, anonymity and confidentiality of information were assured. The Medical Ethics Committee of Tianjin University of Traditional Chinese Medicine evaluated and approved this work, and the researchers reviewed the study in detail with the subjects and ensured that they had no objections to the study. It was based on voluntary participation and was conducted anonymously after participants signed an informed consent form. There were no negative consequences for elderly who refused to participate in this study. All study participants were informed that their responses were confidential, as was their anonymity.

## 3 Materials

### 3.1 Elder psychological abuse scale (EPAS)

Liu (2022) developed the Assessment Elder psychological abuse scale in domestic setting. This scale includes 16 items across the following four domains: (1) disrespect (e.g., “Family members speak to you in a tone that causes discomfort, such as shouting or showing impatience”), (2) isolation (“You are ignored by family members at their suggestion”), (3) threat (“Your family threatens to confiscate or damage your assets e.g., bank deposits, real estate”), and (4) control (“Family members deprive you of basic necessities including food, clothing, medication or medical care”). A 5-point Likert scale was used, where 1 = “none,” 2 = “rarely,” 3 = “sometimes,” 4 = “often,” and 5 = “always.” The total score, calculated by summing all item scores, provides a quantitative measure of psychological abuse severity, with higher scores indicating more severe abuse experiences. The Cronbach's α for the scale in the Liu study was 0.788, and the test-retest reliability was 0.954 (Liu, 2022).

### 3.2 Brief aging perceptions questionnaire (B-APQ)

The brief aging perceptions questionnaire, originally utilized by Professor Sexton in 2014 to assess the perceived degree of aging among older adults (Sexton et al., 2014). Later, Hu et al. (2018) translated it into Chinese for our country. The scale includes 5 dimensions, including negative outcomes and control, positive outcomes, chronic time, positive control, and affective representation, with a total of 17 entries. Entry options were rated on a 5-point Likert scale from “Strongly Disagree” to “Strongly Agree,” with scores ranging from 1–5 (entries 4, 5, 6, 8, 9, and 10 were reverse scored), and the sum of all entries was the total scale score, which ranged from 17 to 85 points. The total score ranges from 17 to 85. The higher the total score, the more negative the attitude of older adults toward self-perceived aging. The scale was confirmed to be reliable, with a Cronbach's α coefficient of 0.914 (Hu et al., 2018).

### 3.3 Emotion regulation scale (ERS)

The scale, developed by Wang et al. (2007) contains 14 entries and 2 dimensions. Seven items measure the cognitive reappraisal dimension (questions 2, 5, 7, 9, 10, 12, and 13) and seven items measure the expressive inhibition dimension (questions 1, 3, 4, 6, 8, 11, and 14). The scale was scored on a 7-point Likert scale varying from 1 to 7, with 1 indicating complete disagreement and 7 indicating complete agreement. Higher scores indicate that individuals are most likely to use appropriate emotion regulation strategies. The Cronbach's α coefficient for cognitive reappraisal was 0.83, and the Cronbach's α coefficient for expressive inhibition was 0.77 (Wang et al., 2007).

## 4 Data analysis

Data analysis was performed using SPSS 26 and AMOS 26 software programs. Pearson's correlation analysis was used to explore the relationships among self-perceived aging, cognitive reappraisal, expressive inhibition and psychological abuse.

Structural equation methodology is a confirmatory factor analysis used to study relationships between hidden variables. Each hidden variable is influenced by numerous observed variables (dimensions). Cognitive reappraisal and expressive inhibition scores on the two dimensions of emotion regulation, and scores on each dimension of self-perceived aging and psychological abuse were used as measurement variables (indicator variables). The combined self-perceived aging and psychological abuse scores were used as the organizational variable (latent variable). The study used the maximum likelihood method to estimate the covariance matrix and the bias-corrected percentile bootstrap test to evaluate direct and indirect effects, with 2,000 bootstrap samples. Additionally, Byrne's invariance testing strategy was adopted to cross-validate the model using the chi-squared difference test for post model fitting issues. A good fitting model needed to meet the following recommendations: chi-square/degrees of freedom (χ^2^/*df* < 3), normed fit index (NFI > 0.90), goodness-of-fit index (GFI > 0.90), standardized root mean square residual index (SRMR < 0.05) and root mean square error of approximation index (RMSEA < 0.08).

## 5 Results

### 5.1 Descriptive results and preliminary analysis

In this study, a total of 449 questionnaires were collected. Questionnaires with missing values in items, missing important information, recognizable patterns in the scale answers, or unchanged answers across several consecutive questions were excluded. A total of 14 invalid questionnaires were removed, and 435 valid questionnaires remained, resulting in an effective response rate of 96.88%. Table 1 shows the means, standard deviations of the demographic characteristics of the participants and variables.

**Table 1:** Characteristics of the participants (*n* = 449).

**Elder characteristics**	***M***	***SD***
**Gender**		
Male	163	37.50
Female	272	62.50
**Education level**		
Primary and below	251	57.70
Junior high school	109	25.10
Senior high school	61	14.00
College degree and below	14	3.20
**Marital status**		
Married	338	77.70
Divorcee	4	0.90
Live apart	2	0.50
Bereaved of one's spouse	89	20.50
Remarry	2	0.50
**Residency**		
Live alone	47	10.80
Living with spouse only	207	47.60
Living with child's family only	86	19.80
Living with spouse and children's family	95	21.80
**Monthly salary(**¥**)**		
< 800	160	36.80
801–1500	98	22.50
1501–2500	45	10.30
2501–3500	50	11.50
3501–4500	40	9.20
>4500	42	9.70
**Chronic disease**		
Yes	330	75.90
No	105	24.10
**Frequency of participation in community activities**		
Often	20	4.60
Sometimes	84	19.30
Occasionally	43	9.90
Rarely	288	66.20
**Age (years)**	69.39	7.10
**Self-perceived aging**	2.84	0.59
Negative outcomes and control	3.53	0.66
Positive outcomes	2.58	1.08
Chronic time	4.18	0.98
Positive control	1.87	0.86
Affective representations	1.60	0.86
**Emotion regulation**	4.74	0.78
Cognitive reappraisal	5.66	0.82
Expressive inhibition	3.83	1.41
**Psychological abuse**	1.64	0.38
Disrespect	1.85	0.66
Isolation	1.82	0.34
Threats	1.02	0.11
Control	1.62	0.66

Pearson's correlation coefficient test was used to explore the correlation between self-perceived aging scores and dimensions of emotional regulation and psychological abuse in older adults. The correlation between self-perceived aging and the dimensions of emotion regulation was significant (*P* < 0.001) and significantly correlated with the dimensions of psychological abuse (*P* < 0.01). Cognitive reappraisal, expressive inhibition and some dimensions of psychological abuse are significantly correlated (Table 2).

**Table 2:** Correlation analysis between the studied variables (*N* = 449).

**Variables**	**2**	**3**	**4**	**5**	**6**	**7**	**8**	**9**	**10**	**11**	**12**	**13**	**14**
1	0.063	0.254^**^	0.749^**^	0.698^**^	0.719^**^	0.668^**^	0.531^**^	−0.321^**^	0.205^**^	0.219^**^	0.182^**^	0.170^**^	0.152^**^
2		0.014	0.093	0.005	0.01	0.049	0.101^*^	0.262^**^	0.872^**^	0.023	−0.017	−0.034	0.020
3			0.09	0.110^*^	0.236^**^	0.157^**^	0.298^**^	−0.203^**^	0.132^**^	0.914^**^	0.724^**^	0.169^**^	0.773^**^
4				0.357^**^	0.492^**^	0.431^**^	0.295^**^	−0.192^**^	0.180^**^	0.062	0.077	0.156^**^	0.068
5					0.363^**^	0.421^**^	0.206^**^	−0.296^**^	0.144^**^	0.118^*^	0.061	0.110^*^	0.009
6						0.358^**^	0.235^**^	−0.282^**^	0.146^**^	0.206^**^	0.153^**^	0.154^**^	0.176^**^
7							0.215^**^	−0.230^**^	0.133^**^	0.149^**^	0.073	0.157^**^	0.049
8								−0.121^*^	0.146^**^	0.269^**^	0.214^**^	0.015	0.228^**^
9									−0.182^**^	−0.195^**^	−0.131^**^	−0.137^**^	−0.127^**^
10										0.131^**^	0.062	0.070	0.106^*^
11											0.494^**^	0.159^**^	0.601^**^
12												0.095^*^	0.488^**^
13													0.055
14													

1, Self-perceived aging; 2, Emotion regulation; 3, Psychological abuse; 4, Negative outcomes and control; 5, Positive outcomes; 6, Chronic time; 7, Positive control; 8, Affective representations; 9, Cognitive reappraisal; 10, Expressive inhibition; 11, Disrespect; 12, Isolation; 13, Threats; 14, Control.

^*^p < 0.050. ^**^p < 0.001.

### 5.2 Test of mediation effects

**Figure 1:**
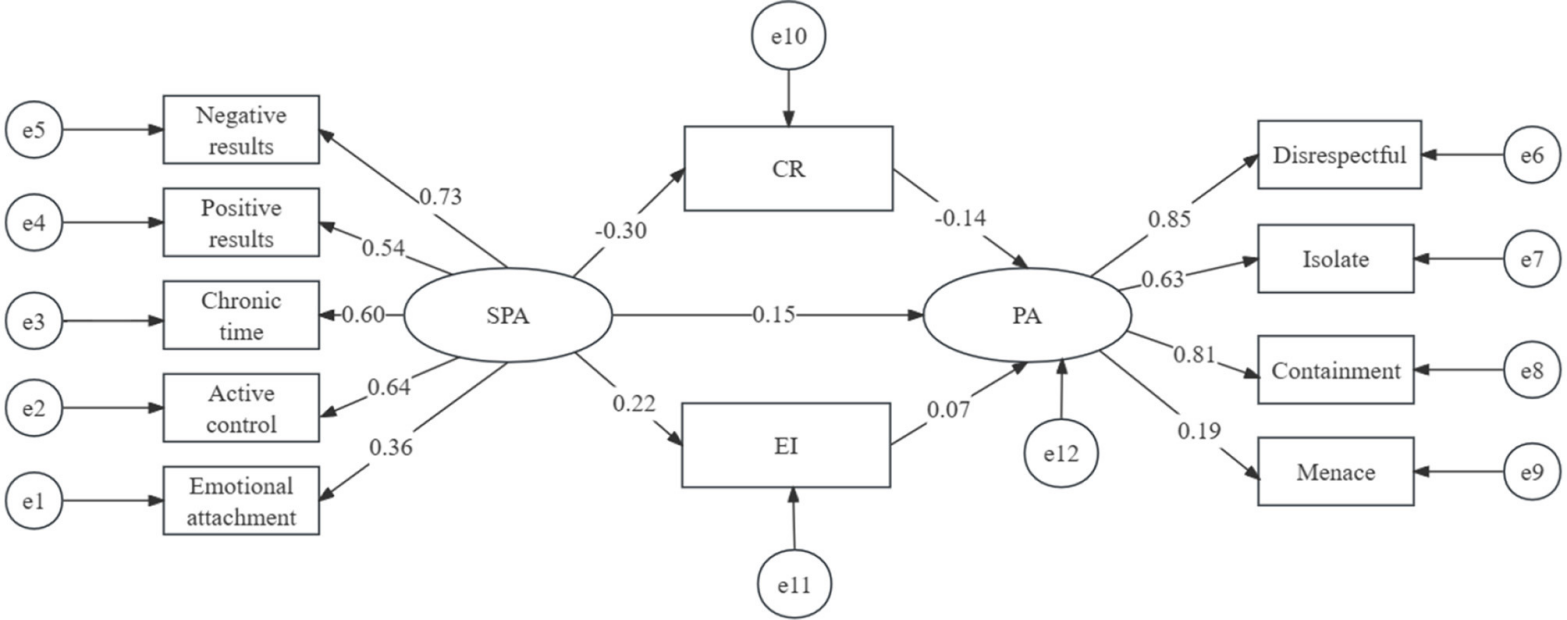
Final model and standardized model paths between cognitive reappraisal and expressive inhibition on elderly' self-perceived aging and psychological abuse. SPA, self-perceived aging; PA, psychological abuse; CR, cognitive reappraisal; EI, expressive inhibition.

In order to examine the relationship between self-perceived aging and psychological abuse and the mediating role of cognitive reappraisal and expressive inhibition, the researchers conducted path analysis and structural equation modeling (SEM) using AMOS software. For this reason, the structural model in Figure 1 was tested to understand the relationships among the four variables.

The mediation analysis tested a hypothetical model, which was subsequently modified to improve model fit. The structural equation modeling demonstrated excellent fit indices (χ^2^/*df* = 2.807, RMSEA = 0.065, CFI = 0.992, TFI = 0.896, IFI = 0.923, NFI = 0.886). The hypothesized model showed that self-perceived aging affects the mediating variables of cognitive reappraisal and expressive inhibition, which in turn influence the psychological abuse variable (Figure 1). The overall effect of self-perceived aging on psychological abuse was significant (β = 0.207, SE = 0.22, *P* < 0.01).The total mediating effect of the two mediating factors was statistically significant [β = 0.055, SE = 0.072, 95% CI (0.055, 0.339)], and the mediating pathway of cognitive reappraisal was statistically significant (β = 0.042, *P* = 0.007), but the mediating pathway effect of expression repression was not significant (β = 0.015, *P* = 0.139). In other words, self-perceived aging can influence psychological abuse indirectly through cognitive reappraisal and simultaneously through cognitive reappraisal and expression suppression. Furthermore, cognitive reappraisal and expressive inhibition together explained 27% of the variance in psychological abuse among older adults. Tables 3, 4 summarize the results of the multiple mediating effects.

**Table 3:** Elderly people's self-perceived aging, cognitive reappraisal, expressive inhibition, and psychological abuse pathway coefficients(*N* = 449).

**Pathway**			**Standard coefficient**	**SE**	**CR**	***P* value**
CR	←	SPA	−0.297	0.429	−4.322	< 0.001
EI	←	SPA	0.223	0.681	3.532	< 0.001
PA	←	CR	−0.137	0.027	−2.508	0.012
PA	←	EI	0.067	0.015	1.256	0.209
PA	←	SPA	0.152	0.210	2.220	0.026
Affective representations	←	SPA	0.356	0.412	14.038	< 0.001
Active control	←	SPA	0.644	0.296	6.139	< 0.001
Chronic time	←	SPA	0.602	0.321	6.029	< 0.001
Positive results and result	←	SPA	0.543	0.330	5.840	< 0.001
Negative results	←	SPA	0.726	0.413	6.276	< 0.001
Disrespectful	←	PA	0.848	0.499	6.179	< 0.001
Isolate	←	PA	0.625	0.031	12.293	< 0.001
Containment	←	PA	0.811	0.040	14.264	< 0.001
Menace	←	PA	0.187	0.006	3.582	< 0.001

SPA, self-perceived aging; CR, Cognitive reappraisal; EI, Expressive inhibition, PA, Psychological abuse.

**Table 4:** Total effects, direct effects, and indirect effects of cognitive reappraisal and expressive inhibition on elderly self-perceived aging and psychological abuse(*N* = 449).

					**95% CI**	
**Project**	**Variables**	β	**SE**	***P***	**LB**	**UB**
Total effect	SPA on PA	0.207	0.220	0.004	0.240	1.092
Direct effect	SPA on PA	0.152	0.222	0.029	0.044	0.936
Total indirect effect	SPA on PA	0.055	0.072	0.004	0.055	0.339
Indirect effect	CR	0.042		0.007	0.032	0.283
Indirect effect	EI	0.015		0.139	−0.018	0.154

SPA, Self-perceived aging; CR, Cognitive reappraisal; EI, Expressive inhibition, PA, Psychological abuse.

## 6 Discussion

Given the severe physiological, mental, and social consequences of psychological abuse for elderly people. Considering that social security and elderly care resources in rural China are less developed than in urban areas-thereby elevating the vulnerability of rural elderly people to psychological abuse (Wu et al., 2025). This study focuses on this particularly underserved group. It aims to explore the impact of self-perceived aging on psychological abuse in elderly people and the underlying mechanism. As expected, the results of this study confirm that self-perceived aging is related to psychological abuse (Hypothesis 1) and further illustrate that cognitive reappraisal and expressive inhibition mediates the relationship between self-perceived aging and psychological abuse (Hypothesis 2, 3).

### 6.1 The direct impact of self-perceived aging on psychological abuse

Self-perceived aging is one of the key predictors of successful aging and physical and psychological health (Li et al., 2023). Previous research has shown that older adults who are more physically active have more positive perceptions of aging (Beyer et al., 2015). The results of the present study are in agreement with Tovel's findings. The more positive SPA is, the stronger the self-efficacy beliefs are. Stronger self-efficacy beliefs may motivate older people to adopt more effective coping styles and encourage them to seek help, thus reducing the incidence of psychological abuse (Tovel et al., 2019). Older adults with negative SPA may perceive themselves as burdens to their families. This phenomenon is particularly pronounced in rural areas, where factors such as geographic isolation, inadequate social services, and limited public awareness of elderly rights protection collectively contribute to higher psychological abuse. As a result, it becomes extremely challenging for older adults who have experienced psychological abuse to seek social support, which is crucial for preventing the recurrence of such abuse (Hu and Li, 2022).

### 6.2 Mediating role of cognitive reappraisal and expression inhibition

Self-perceived aging predicts psychological abuse through the mediating mechanism of cognitive reappraisal, although this mediating role remains under recognized. Successful emotion regulation protects not only the physical health of older adults but also their mental resilience. Recent research by Sakakibara suggests that the mediating role of cognitive reappraisal in perceived aging in older adults can increase wellbeing (Löckenhoff and Carstensen, 2004; Sakakibara and Ishii, 2020). More old adults tend to develop richer positive coping strategies and use cognitive reappraisal more frequently than younger adults. This age-related emotional regulation pattern enhances positive affect while attenuating negative emotions, thereby reducing vulnerability to psychological abuse.

Mediating effects of emotion regulation can be explained by the SST that an individual who is successfully aging has high physical and cognitive function. Elderly people employ emotion regulation strategies to enhance psychological wellbeing, aiming to maximize positive emotional experiences while minimizing affective risks following psychological abuse (John and Gross, 2004; Soylu and Ozekes, 2023). Positive SPA contributed to older adults' utilization of emotion regulation strategies. Specifically, a positive aging outlook enhances seniors' confidence in their ability to regulate emotions, which may consequently reduce adverse outcomes associated with psychological abuse. People who have suffered abuse have difficulty regulating their emotions after a traumatic experience. They use psychologically avoidant coping styles and are unable to be purpose-oriented. This situation thus triggers feelings of helplessness and out-of-control behavior (Siegel and Lahav, 2022; Villalba et al., 2023). In a study on child abuse, an intriguing phenomenon came to light. Among those who had endured abuse, some had the experience of receiving emotional regulation guidance. These individuals actively employed cognitive reappraisal strategies. Through this approach, they were able to effectively harness the emotion control network to regulate the amygdala, which is highly sensitive to negative emotions (McLaughlin et al., 2015). Other studies have shown the same results, revealing stronger amygdala connections in frontal and parietal regions during psychological observations of abused adults. Demonstrating that effective emotion regulation, complemented by sharp frontal limbic regulation, may support resilience after abuse (Demers et al., 2022).

Expressive inhibition, a negative subset of emotion regulation strategies, may render elderly individuals with negative SPA more vulnerable to adversity, which may increase the risk of psychological abuse (Yu et al., 2023). A meta-analysis by Rogier et al. (2024) showed that expressive inhibition is strongly associated with suicide, which is particularly prominent among older adults. This finding suggests that expressive inhibition strategies not only affect self-perceived aging, but may also increase the risk of more serious psychosocial risks. Research shows that self-perceived aging influences an individual's choice of emotion regulation strategies (Gurera et al., 2022; Bellingtier et al., 2022). The analysis suggests that individuals with negative SPA find it difficult to recognize and express emotions, leading them to over focus on external matters and making them more susceptible to more negative cognitions and worst coping strategies. This handicap can trigger maladaptive cognitive emotion regulation strategies such as expressive inhibition, which may further lead to negative SPA and pessimism. However, in this study expression inhibition had no mediating effect. This is likely because expressive inhibition strategies come into play after an emotion has emerged. The strategy only lessens an individual's expression of negative emotions but fails to alter their perception of the abusive event. In fact, in some instances, it may even heighten the individual's negative experiences and induce dysphoria. Previous research has shown that individuals who have experienced abuse are more inclined to use the strategy of expressive inhibition compared to others (Garnefski et al., 2005).

### 6.3 Implications and limitations

The current study has important practical implications for preventing psychological abuse of elderly people in rural China. First, this study draws upon Socioemotional Selectivity Theory (SST) to clarify the crucial role of positive SPA in reducing psychological abuse risk. Secondly, the findings of this study demonstrate that cognitive reappraisal plays a crucial mediating role. Integrating cognitive reappraisal techniques into clinical interventions, such as Emotion-Focused Therapy (EFT), could help individuals enhance stress management capacity, improve psychological resilience post-abuse, and mitigate the adverse effects of psychological distress. Furthermore, psychological educators should prioritize strengthening emotion regulation skills among this vulnerable population to bolster their psychological defenses against abuse. The findings of this study propose a novel direction for clinical practice by integrating self-perceptions of aging with emotion regulation strategies. The study provides actionable implementation pathways for preventing psychological abuse among rural elderly populations.

This study also has limitations which could be further improved in the future research. The first limitation is the cross-sectional design, which is not perfect enough to draw clear conclusions in terms of causal effect, for it only suggests a certain causal ordering in the relationships tested. The future study can use a longitudinal design. In addition, the collected data were based on self-reporting (i.e., self-perceived aging, cognitive reappraisal, expressive inhibition), which might be subject to bias. Also, this study was conducted in a Chinese cultural context, and caution should be taken to generalize the results to groups of older people in different countries or contexts.

## 7 Conclusions

In this study, we found that self-perceived aging was related to psychological abuse, and this relationship was mediated by cognitive reappraisal and expressive inhibition. In addition to this, our study utilized the SST to validate the effects of self-perceived aging, emotion regulation on psychological abuse, and to further explain the study variables. In terms of practical implications, this study reveals the importance of positive SPA attitudes and cognitive reappraisal strategies in reducing psychological abuse. Theory-driven actions by family members, community workers, and health care providers should be encouraged to reduce the incidence of psychological abuse among older adults in rural China.

## Data Availability

The datasets presented in this article are not readily available, as they contain patient-sensitive information. Researchers who require access to this data may contact the corresponding author (douhaoying11@126.com), to ensure proper data handling under controlled conditions.
